# Urbanization data of Samara city, Russia

**DOI:** 10.1016/j.dib.2016.01.056

**Published:** 2016-02-03

**Authors:** Mukesh Singh Boori, Komal Choudhary, Alexander Kupriyanov, Viktor Kovelskiy

**Affiliations:** a Samara State Aerospace University, Samara, Russia; b American Sentinel University, CO, USA; c Hokkaido University, Sapporo, Japan

**Keywords:** Urban expansion, Land use/cover change detection, Remote sensing & GIS

## Abstract

A method has been developed for urbanization by using satellite data and socio-economic data. These datasets consists three decade Landsat images and population data. A detailed description using flow chart is given to show how to use this data to produce land use/cove maps. The land use/cove maps were used to know the urban growth in Samara City, Russia.

**Table:** 

**Specifications table**	
Subject area	Geography
More specific subject area	Remote Sensing and GIS
Type of data	Satellite image, figure, graph
How data was acquired	Collect from field and download from NASA and USGS website
Data format	Img, Tif, Jpg
Experimental factors	Image processing
Experimental features	Image classification, combined satellite data and population data in GIS with the help of ArcGIS software
Data source location	Scientific Research Laboratory of Geo-informatics and Information Security (SRL-55), Samara State Aerospace University, Russia
Data accessibility	All data is in this data article

Value of the data

• Land use/cover data is utilize in maximum type of remote sensing data applications such as hydrology, agriculture, forest, urban growth, vulnerability, natural resources etc.
• Socio-economic or secondary data such a population data is useful to verify the satellite data and to know the growth of an area.
• Data of urban expansion, land use/cover is very useful to local government and urban planners for the future plans to sustainable development of the city.

## 1 Data

Following multi-temporal and multi-spectral satellite data were used: Landsat 5 TM (Thematic Mapper) for 1985 and 1995, Landsat 7 ETM+ (Enhanced Thematic Mapper plus) for 2005 and Landsat 8 OLI (Operational Land Imager) for 2015, an image captured by a different type of sensor. All data were downloaded free of cost from NASA and USGS website. In secondary data we used population data of samara city for last three decades.

## 2 Experimental design, materials and methods

**Fig. 1:**
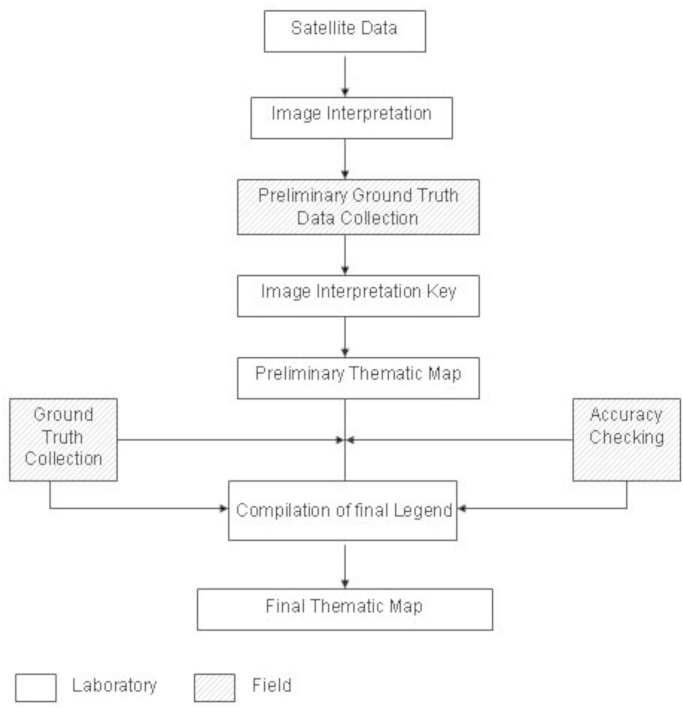
Methodological flow chart.

In methodological part all satellite data go through preprocessing, first use geometric correction, band ratio, than classification and in last change detection (Fig. 1). All four satellite images were classified through maximum likelihood supervised classification in ArcGIS 10.1 software [1], [2]. Also use secondary data such as field data and socio-economic/population data.

**Fig. 2:**
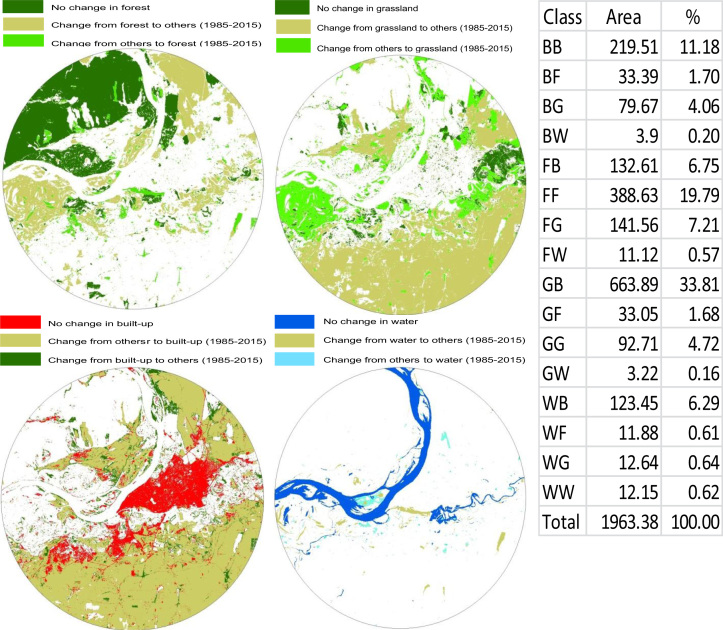
Land use/cove change detection image data maps from 1985 to 2015. [B: Built-up, F: Forest, G: Grassland, W: Water body].

After preprocessing and classification, land use/cover change detection and a post-classification detection method was employed [3], [4]. A pixel-based comparison was used to produce change information on pixel basis and thus, interpret the changes more efficiently taking the advantage of ‘‘-from, -to’’ information (Fig. 2). Classified image pairs of two different decade data were compared using cross-tabulation in order to determine qualitative and quantitative aspects of the changes for the period of 1985–2015. After classification, four major land cover classes were found: forest, built-up, water and grassland. A change matrix [5] was produced with the help of ArcGIS software. Quantitative areal data of the overall land use/cover changes as well as gains and losses in each category between 1985 and 2015 data were then compiled [6].

### 2.1 Urban expansion

**Fig. 3:**
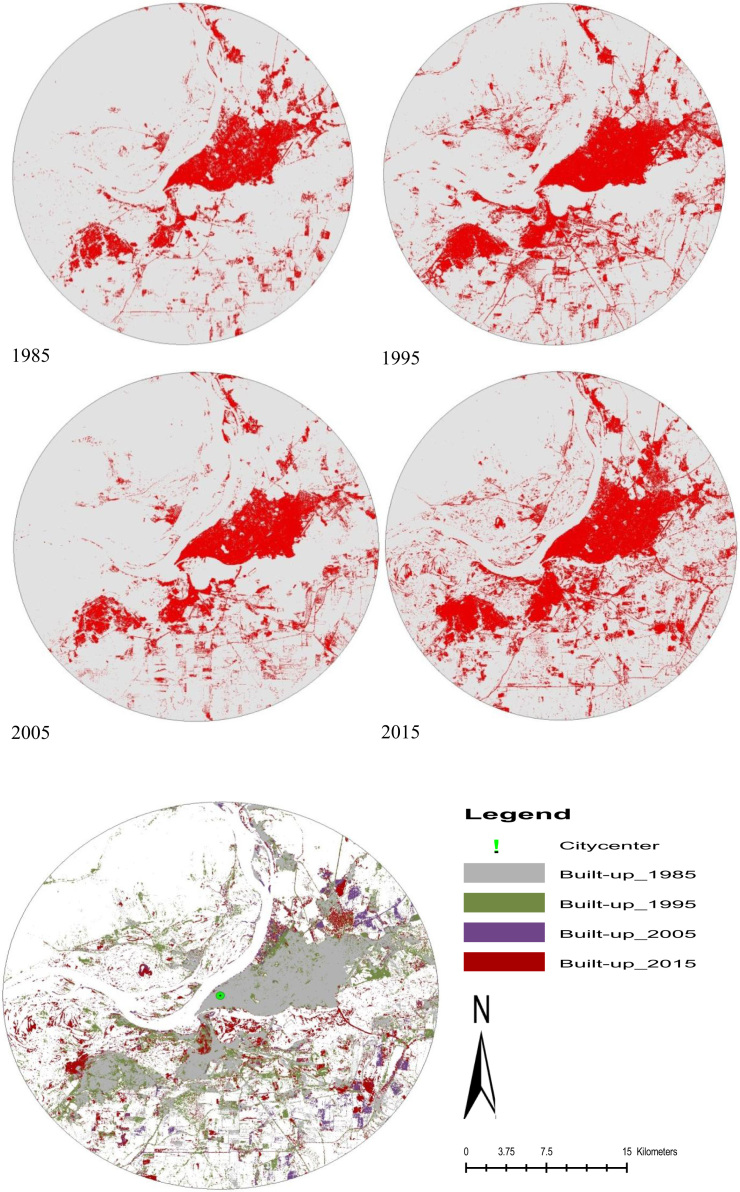
City growth image data maps in different years from 1985 to 2015.

Urban expansion rate and its dynamic change of the spatial structure of a city vary in a temporal sequence. The dynamism of land use class represents change in quantity of a certain land use class in a unit time [7], so this a key index for evaluating spatial change of urban expansion (Fig. 3). By analyzing the dynamism of land use, the extent and rate of urban expansion can be compared quantitatively [8], according to the following formula and produce urbanization data maps (Fig. 3):

(1) $$\mathrm{LUDI}=\frac{\mathrm{Ua}-\mathrm{Ub}}{\mathrm{Ua}}\times \frac{1}{T}\times 100\%$$

where *Ua* and *Ub* denote areas of a certain land use class at time *a* and time *b* respectively; *T* denotes the length of time from time *a* to time *b*. When *T* is in a unit of year, then LUDI is the annual rate of change in area for this land use class.

**Fig. 4:**
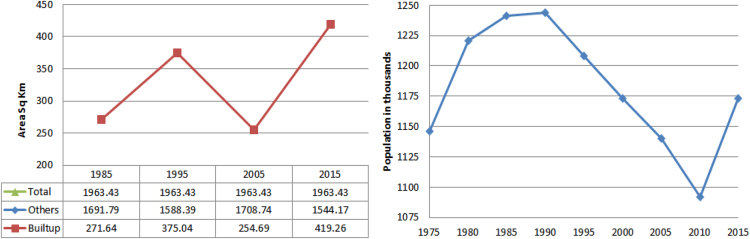
Population and built-up area change graph.

This data explores the spatial-temporal pattern of land use/cove change with applicability and effectiveness of satellite data with socio-economic data. This data show urban expansion with fast economic development of Samara city, Russia. Compiegne of satellite data with ground truth and population data shows similarity (Fig. 4). It is prove the accuracy of satellite data and its analysis work.

## References

[1] M.S. Boori, V. Vozenilek, Region: a remote sensing and GIS based approach. SPIE Remote SensingLand-cover disturbances due to tourism in Jeseniky mountain, 9245, 92450T, 2014, 01–11 doi: 10.1117/12.2065112.

[2] Ye Y., Zhang H., Liu K., Wu Q. (2013). Research on the influence of site factors on the expansion of construction land in the Pearl River Delta, China: By using GIS and remote sensing. Int. J. Appl. Earth Observ. Geoinf..

[3] Boori M.S., Vozenilek V., Choudhary K. (2015). Land use/cover disturbances due to tourism in Jeseniky Mountain, Czech Republic: a remote sensing and GIS based approach. Egypt. J. Remote Sens. Space Sci..

[4] Shu B., Zhang H., Li Y., Qu Y., Chen L. (2014). Spatiotemporal variation analysis of driving forces of urban land spatial expansion using logistic regression: a case study of port towns in Taicang city, China. Habitat Int..

[5] Boori M.S., Vozenilek V., Burian J. (2014). Land-cover disturbances due to tourism in Czech Republic.

[6] Boori M.S., Amaro V.E. (2010). Land use change detection for environmental management:using multi-temporal, satellite data in Apodi Valley of northeastern Brazil. Appl. GIS.

[7] Hu Z.L., Du P.J., Guo D.Z. (2007). Analysis of urban expansion and driving forces in Xuzhou City based on remote sensing. J. China Univ. Min. Technol..

[8] Boori M.S., Ferraro R.R. (2015). Global Land Cover classification based on microwave polarization and gradient ratio (MPGR).

